# Targeting the RNA-Binding Site of SARS-CoV‑2 NSP13 by FRASE-bot in CACHE Challenge #2

**DOI:** 10.1021/acs.jcim.6c00560

**Published:** 2026-08-06

**Authors:** Xiaowen Wang, Akhila Mettu, Oleksandra Herasymenko, Madhushika Silva, Scott Houliston, Irene Chau, Pegah Ghiabi, Elisa Gibson, Sumera Perveen, Almagul Seitova, Matthieu Schapira, Dmitri Kireev

**Affiliations:** † 201642 University of Missouri, Department of Chemistry, Columbia, Missouri 65211, United States; ‡ Structural Genomics Consortium, 7938 University of Toronto, Toronto, Ontario M5G 1L7, Canada; § Princess Margaret Cancer Centre, 7989 University Health Network, Toronto, Ontario M5G 2C4, Canada; ∥ Department of Pharmacology & Toxicology, University of Toronto, Toronto, Ontario M5S 1A8, Canada

## Abstract

The Critical Assessment of Computational Hit-Finding Experiments (CACHE) Challenges came forward as real-world stress tests for virtual screening methods. In Challenge #2, the focus was on SARS-CoV-2 NSP13 helicase—the most conserved protein across Coronaviridae and a prime target for broad-spectrum antivirals. We brought in FRASE-based hit-finding robot (FRASE-bot), our fragment-based screening engine that delimits target-relevant chemical space and identifies likely binding sites. Guided by FRASE-bot, 79 compounds were selected over two rounds. Round 1 delivered four confirmed binders of NSP13 with dissociation constants between 11 and 61 μM. Round 2 pushed further: five analogs bound in the micromolar range, with compounds **8** and **9** (derived from the parent hit **2**) showing clear gains over other CACHE participants. With successful runs in both CACHE #1 and #2, FRASE-bot is proving its ability to tackle tough protein targets. The identification of validated binders targeting the RNA-binding groove of SARS-CoV-2 NSP13 holds significant promise for antiviral drug discovery.

## Graphical abstract

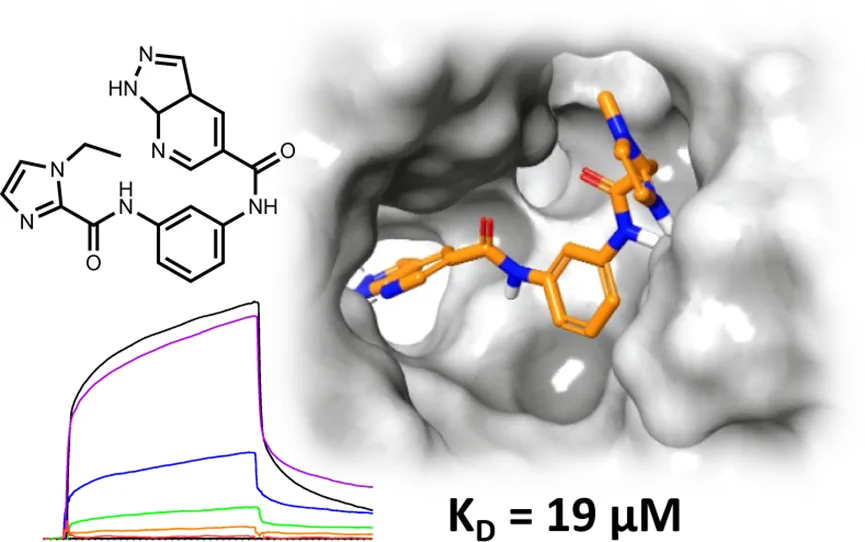

## Introduction

The Critical Assessment of Computational Hit-Finding Experiments (CACHE) Challenges are a public–private initiative to benchmark computational approaches for hit identification against predefined target proteins.^1^ Applicants for the Challenges highly ranked by their peers are invited to submit up to 100 screened compounds for procurement and experimental confirmation. For the first time, these Challenges offer a unique chance to see how virtual screening methods perform outside curated case studies—revealing their true value in head-to-head comparisons on difficult, real targets.

In the second CACHE Challenge (CACHE #2), the SARS-CoV-2 nonstructural protein 13 (NSP13) helicase was selected as the target protein.^2^ As of January 2025, COVID-19 had caused over 777 million confirmed infections worldwide and more than seven million deaths,^3^ underscoring the continued need for broad-spectrum antiviral strategies. NSP13 unwinds viral RNA in the presence of ATP and acts as a viral interferon antagonist, playing a key role in replication.^4–6^ Crucially, it is also the most conserved protein across the coronavirus family, making it a particularly compelling therapeutic target.^7^

The RNA-binding channel offers a more conserved alternative to the canonical nucleotide-binding site. Structural studies revealed fragments overlapping this channel,^5,8^ and conservation analysis showed that ∼88% of residues are preserved across coronaviruses compared to ∼68% in the ATP site.^5^ For these reasons, CACHE #2 focused on the RNA-binding site as the pocket for hit discovery.

In this study, we applied FRASE-bot^9^ to the SARS-CoV-2 NSP13 helicase, focusing on its RNA-binding site—a particularly challenging pocket for small-molecule discovery. FRASE-bot is an automated hit-finding platform^9^ based on the concept of FRAgments in Structural Environments (FRASE),^10^ which exploits recurring fragment-protein interaction patterns to guide the search for drug-like ligands in large chemical libraries. By reusing structural knowledge, FRASE-bot can effectively narrow down the chemical space to target-relevant compounds. The platform has already proven its value in CACHE Challenge #1, where it was among the winning methods in finding ligands for the WD40 repeat of LRRK2,^11,12^ a therapeutic target for Parkinson’s disease, and in an earlier case study identifying ligands for the calcium- and integrin-binding protein CIB1,^9^ a target for triple-negative breast cancer—both targets considered highly intractable. Applying FRASE-bot to NSP13 thus offered an opportunity to test its capacity to tackle one of the most conserved and elusive viral proteins.

## Results

### FRASE Screening Identifies Ligand Fragments Seeded in the NSP13 RNA-Binding Groove

The initial definition of the target binding site within the extended RNA-binding groove was guided by structural data disclosed by the CACHE challenge organizers. Specifically, we anchored our target region around the footprints of cocrystallized fragments found in four X-ray structures of NSP13 (PDB IDs: 5RLH, 5RLZ, 5RML, and 5RMM^5^). We anticipated that while these experimental fragments provided a reliable starting point, the binding site boundaries would be extended and more precisely mapped on the basis of the structural consensus of hits generated during the subsequent FRASE screening.

We then carried out FRASE database screening, as outlined in ref 9, on the NSP13 helicase crystal structure (PDB ID: 5RLH),^5^ identifying 532 fragments spread over various regions of the target (Figure 1A). Subsequently, the FRASE scoring model, a neural network trained to differentiate true FRASEs from decoys,^9^ was applied to assess fragment relevance. This further narrowed down the FRASE-based fragment list to 76 fragments occupying two distinct regions of the protein with 64 fragments confined to the immediate vicinity of the initial footprint within the RNA-binding groove. Further structural clustering using the K-means method^13^ followed by visual analysis allowed us to reduce the FRASE-based target-relevant fragment space to 14 nonredundant fragments. The selected fragments (Figures 1B and 2A) display significant structural diversity and a broad range of noncovalent interacting groups including aryl-sulfonamide, triazole, purine-amine, indazolinone, and others.

**Figure 1:**
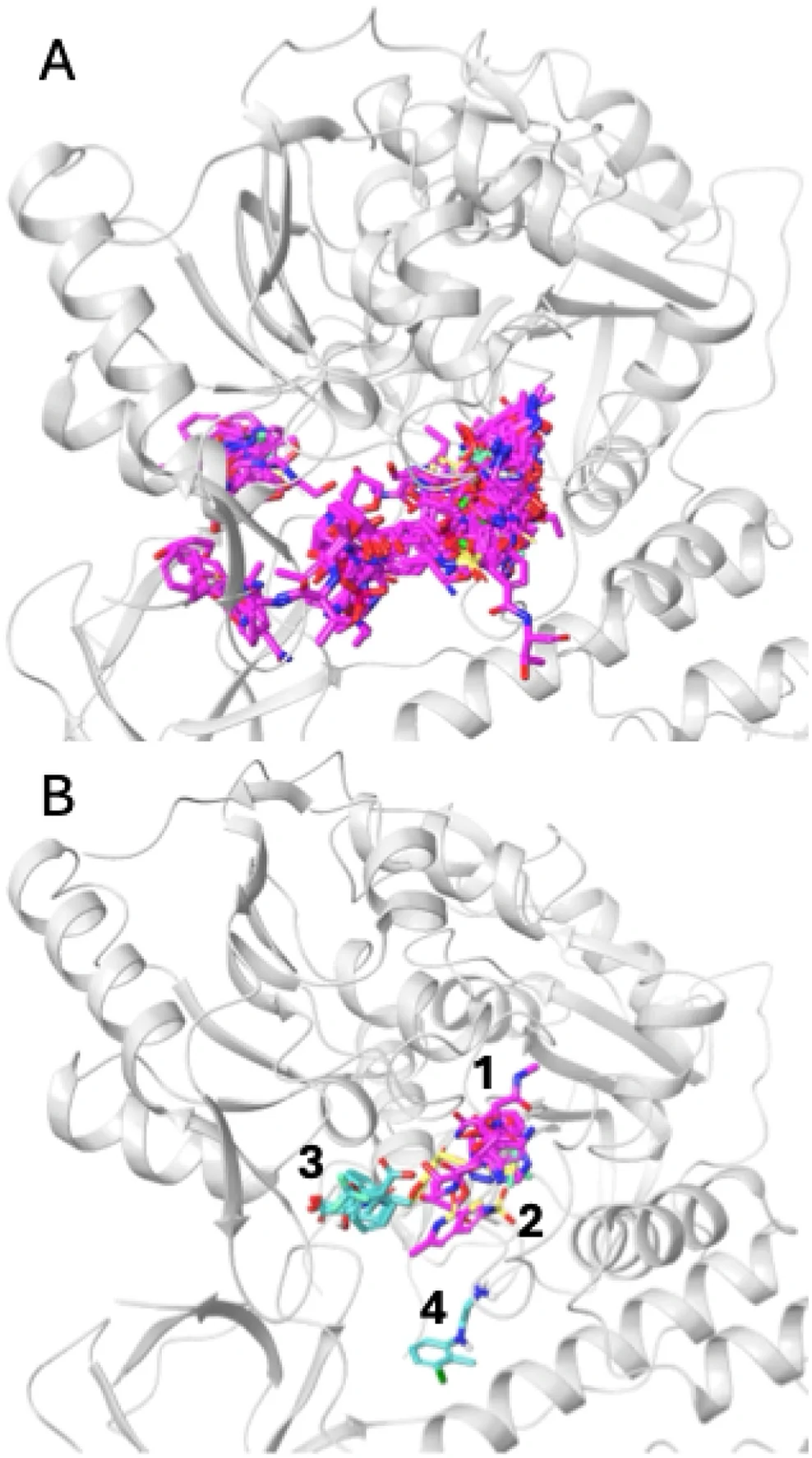
(A) Ligand fragments seeded by the primary FRASE screening (magenta); (B) fragments selected by the FRASE scoring model (magenta) and cocrystallized fragments (cyan); numbers specify spatially distinct fragment clusters.

**Figure 2:**
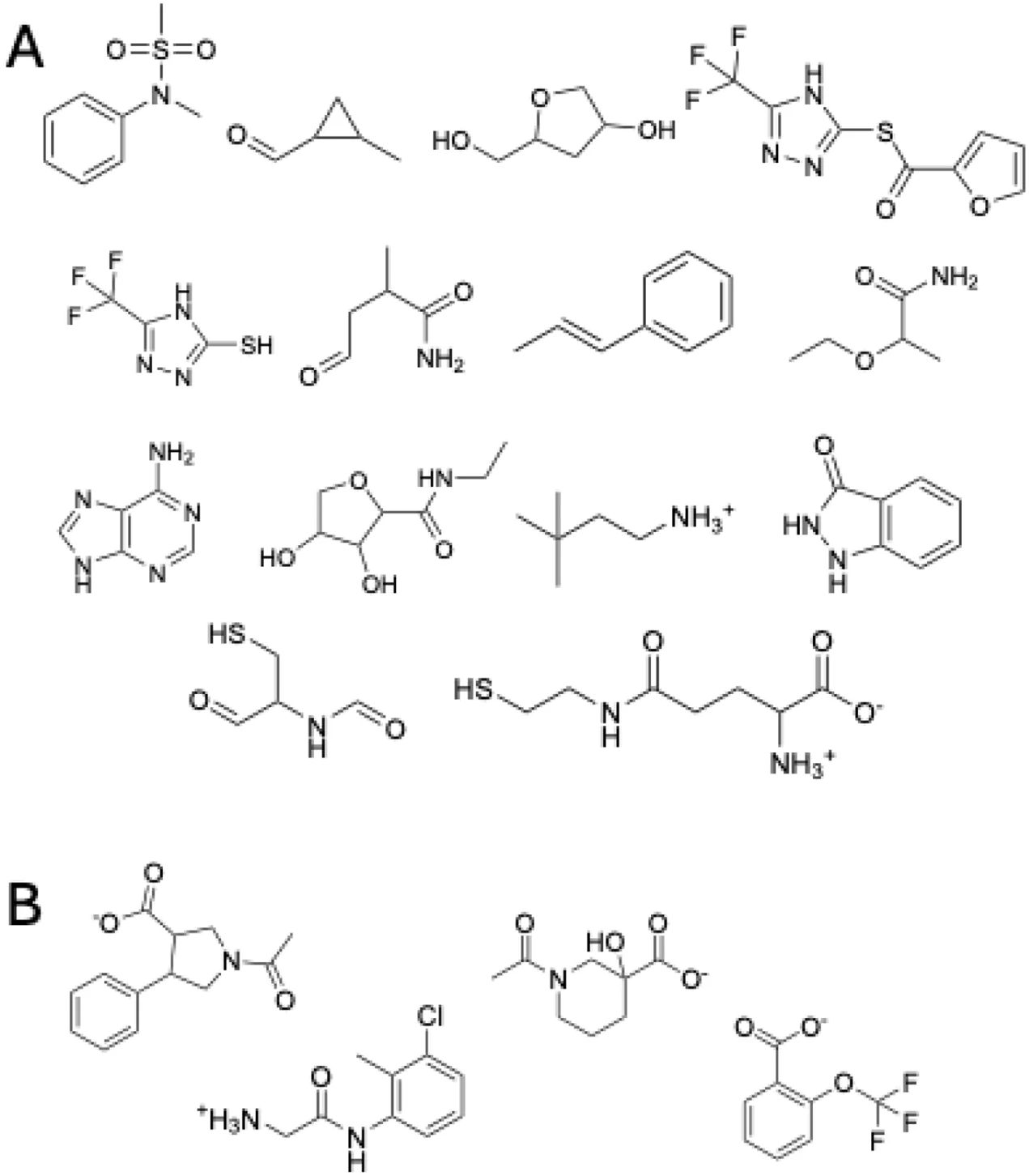
(A) Nonredundant target-specific fragments identified by FRASE screening; (B) cocrystallized fragments (PDB ID: 5RLH, 5RLZ, 5RML, and 5RMM).

### Ligand Fragments Expose Putative Ligand Pockets in the RNA-Binding Site

FRASE screening enables the identification not only of ligand fragments but also of putative binding pockets that can be exploited for structure-based hit finding, effectively mapping the full three-dimensional boundaries of the target site. Our next step was to define the ligand-pocket compartments in the RNA-binding groove that could host distinct parts of a small-molecule ligand and ultimately define the volume for downstream docking. To this end, we applied the *k*-means clustering algorithm^13^ to the geometric centers of the 14 FRASE fragment hits (Figure 2A) and 4 cocrystallized fragments (PDB IDs: 5RLH, 5RLZ, 5RML, and 5RMM)^5^ (Figure 2B).

This clustering served two purposes: first, to define spatially distinct ligand-binding compartments and, second, to guide fragment combination. Fragments belonging to the same cluster were considered mutually exclusive because of spatial overlap, whereas fragments from sufficiently distant clusters were deemed incompatible for direct linkage. The analysis yielded 4 spatially distinct groups: two clusters (**1** and **2**) composed of FRASE fragments and two others (**3** and **4**) derived from the cocrystallized ligands (Figure 1B).

Analysis of the intercluster distances revealed that clusters **1** and **4** are located approximately 14 Å apart, a distance unlikely to be bridged by a single drug-like compound. Conversely, the geometric centers of all other clusters are sufficiently close to be considered compatible for a rational fragment combination strategy.

### Seeded Fragments Enable Selection of a Focused Compound Set

*Substructure Searches* on the Enamine REAL set of 6.5 billion synthesizable compounds were performed using all 18 ligand fragments identified in the previous step (Figure 2), as well as fragment combinations picked from all cluster pairs except 1 and 4. The number of hits returned for both single-fragment and fragment-pair searches ranged from zero to millions, yielding a total of approximately 700 million compounds.

These hits were subsequently processed using a hit prioritization scheme. We favored hits resulting from fragment-pair searches and prioritized rarer hits from single-fragment searches, as these were more likely to possess structural novelty. Specifically, hits resulting from the least prolific queries were given the highest priority, while hits from the most prolific single-fragment queries (those exceeding 10 million hits) were entirely excluded. This prioritization strategy ultimately resulted in a focused set of approximately 26 million hits. Subsequent filters, including Lipinski’s rules,^14^ unwanted substructures, and diversity, allowed us to further reduce the number of selected ligands to 8 million compounds that were then submitted to molecular docking. Unwanted substructures were defined as reactive, toxic, or historically assay-interfering functional groups, utilizing structural rules consistent with the established Rapid Elimination Of Swill (REOS) criteria.^15^

*Pharmacophore-Based Screening* was applied to complement the substructure search and further diversify the target-specific ligand space. In the first step, all 18 fragments (Figure 2) were converted into pharmacophoric features. These features were then clustered based on their types and 3D coordinates using the k-means method.^11^ The cluster centers were subsequently used to compose a total of four pharmacophore hypotheses.

Three hypotheses (RRDNA­(I), RRDNA­(II), RHDNA) incorporated both crystal and FRASE fragments, and one was derived solely from cocrystallized fragments (RRNA) (see Supplementary Figure S1 for 3D pharmacophore geometries superposed with the binding pocket). The features are defined as “R” (aromatic feature), “D” (hydrogen-bond donor), “N” (negative ionizable), “A” (hydrogen-bond acceptor), and “H” (hydrophobic). These four hypotheses were then used to screen approximately 70 million compounds randomly selected from the Enamine REAL data set using the Phase algorithm.^16^ This screening resulted in approximately one million hits for subsequent docking.

### Docking-Based Screening Designates Putative NSP13 Binders

The approximately nine million ligands generated in the previous steps (eight million from substructure search and one million from pharmacophore screening) were submitted to a multistage docking and filtering protocol. To optimize computational resources while mitigating scoring function-specific biases, we employed our standard asymmetric soft consensus approach. First, all nine million ligands were docked to the NSP13 RNA-binding site using AutoDock Vina.^17^ Because Vina is open source, it can be deployed across an unlimited number of CPUs, making it highly scalable for an initial massive screening. Next, approximately 100,000 top-ranked AutoDock Vina hits were selected for docking using Glide. Glide is commercially licensed, and its deployment is constrained by the number of available parallel threads, making it prohibitively slow for docking millions of compounds. By applying Glide exclusively to the top fraction of Vina hits, we ensured that the final selections were top Glide hits that also possessed favorable Vina scores. This sequential framework effectively serves as a soft consensus scoring strategy, ensuring computational feasibility while cross-validating the hits with the two distinct scoring functions.

Next, the approximately 100,000 top-ranked AutoDock Vina hits were selected for docking using Glide.^18^ The resulting 10,000 top-scoring ligands based on Glide results were then submitted to Butina clustering on Morgan fingerprints with a Tanimoto similarity threshold of 0.6. A single ligand with the best Glide docking score was selected from each resulting cluster to ensure chemical diversity. These cluster representatives were subsequently ranked by their docking scores, and the top 350 ligands from this ranked list were retained. These 350 selected cluster representatives were subjected to a visual inspection of their docking poses, a step that incorporated both the Vina and Glide scores.

These 350 cluster representatives were subjected to a visual inspection of their docking poses, a step that incorporated both the Vina and Glide scores. This final manual filtering step resulted in the selection of 79 compounds for procurement from the Enamine catalog and subsequent experimental confirmation in the CACHE #2 Round 1 biophysical assays.

### Binding Confirmation for the Round 1 Hits

Surface Plasmon Resonance (SPR) was used to assess the dissociation constant (*K*_*D*_) of 79 Round-1 compounds with the SARS-CoV-2 NSP13 helicase. Compounds with an adjusted relative response between 0.5 and 2.0 (indicative of specific binding) were selected for further confirmation experiments. The ATPase assay was used to assess the inhibitory potency—in general, no correlation was observed between the SPR and ATPase results. Since we could not discount the possibility that suboptimal binding does not lead to measurable enzymatic inhibition, SPR binding data were used as the primary criterion to advance compounds to Round 2 of the Challenge. Additionally, to exclude nonspecific binders, SPR was also used to assess the affinity to the unrelated protein WDR5. Finally, solubility or potential aggregation issues were evaluated using dynamic light scattering (DLS), where compounds with detected laser power below 80% at 100 μM were flagged but retained until final evaluation by the expert panel.

Four compounds—**1** (CACHE_1421_1), **2** (CACHE_1421_21), **3** (CACHE_1421_33), and **4** (CACHE_1421_62)—were eventually confirmed to bind NSP13 with *K*_*D*_ ranging from 11 μM to 61 μM (Figure 3) and not the unrelated protein WDR5. The DLS analysis showed that **2** remained soluble at concentrations below 100 μM, while **1**, **3**, and **4** exhibited solubilities of up to 200 μM concentrations. These experimentally confirmed hits were thus advanced to Round 2. All compounds submitted for the Round 1 experimental confirmation along with SMILES, docking scores, primary SPR screening data, and *K_D_* values can be found in Supplementary Data Set 1.

**Figure 3:**
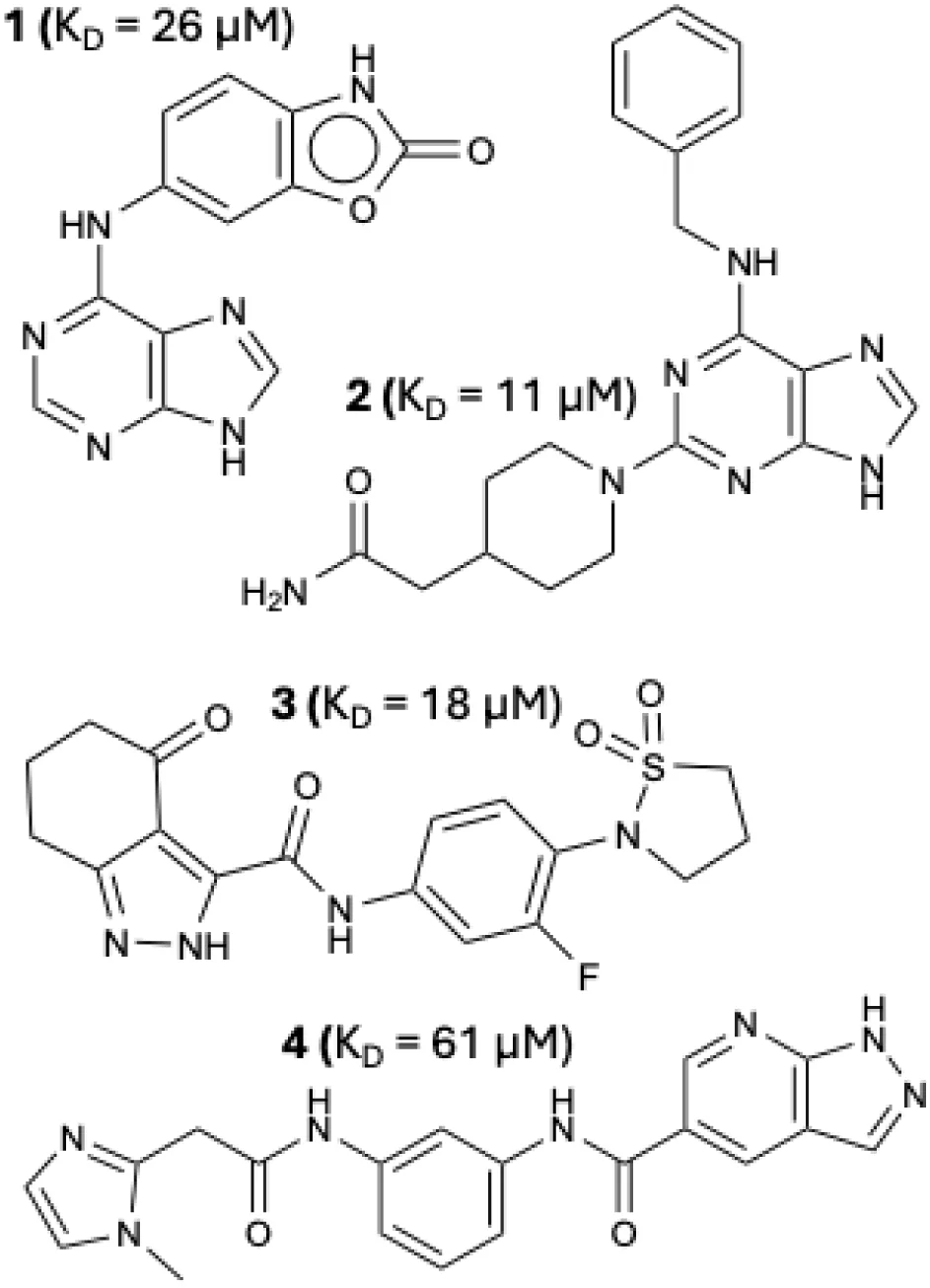
Experimentally confirmed CACHE #2 Round 1 hits. Chemical structures and *K_D_* values were determined by SPR.

### Round 2: Hit Expansion Enables Preliminary Structure–Activity Relationships

CACHE participants qualified for Round 2 were given the opportunity to submit up to 50 structural analogs of the hits identified in Round 1. To this end, we made use of a similarity search in the Enamine REAL 6.5 billion data set followed by Glide docking.^18^ Five hundred analogs with the highest Tanimoto coefficients were retrieved for each of the Round 1 hits **1–4** and docked to the NSP13 RNA-binding pocket. The resulting docking poses were visually inspected to assess their interactions with the target protein—in particular whether they matched the key interactions of their respective parent hits. Based on this evaluation, a total of 37 analogs (14 for **1**, 9 for **2**, 3 for **3**, and 11 for **4**) were retained for procurement and experimental confirmation.

Five analogs were confirmed as NSP13 binders in the Round 2 SPR dose–response assays, showing *K*_*D*_ values ranging from 19 to 98 μM (Figure 4). These include: CACHE2–HO_1421_3 (**5**), an analog of the parent hit **1**; CACHE2–HO_1421_16 (**6**) and CACHE2–HO_1421_12 (**7**), both analogs of **2**; and CACHE2–HO_1421_29 (**8**) and CACHE2–HO_1421_27 (**9**), both analogs of **4**. All analogs except **5** demonstrated some degree of binding to WDR5, though weaker than to NSP13. Additionally, the DLS analysis showed that all analogs were soluble at concentrations of up to 200 μM except the **7** which was soluble at concentrations of up to 100 μM. Finally, analog **5** showed weak inhibitory activity in the ATPase assays (40% inhibition at 50 μM). Overall, only analogs of **4** showed improved binding to NSP13, from 61 μM to 20 μM, while analogs of both **1** and **2** are weaker binders compared to the parent hits. Nevertheless, compounds with varying confirmed affinities are still useful for the analysis of structure–activity relationships and, ultimately, for developing new, more potent ligands. All compounds submitted for the Round 2 experimental confirmation along with SMILES, docking scores, primary SPR screening data, and *K_D_* values can be found in Supplementary Data Set 2.

**Figure 4:**
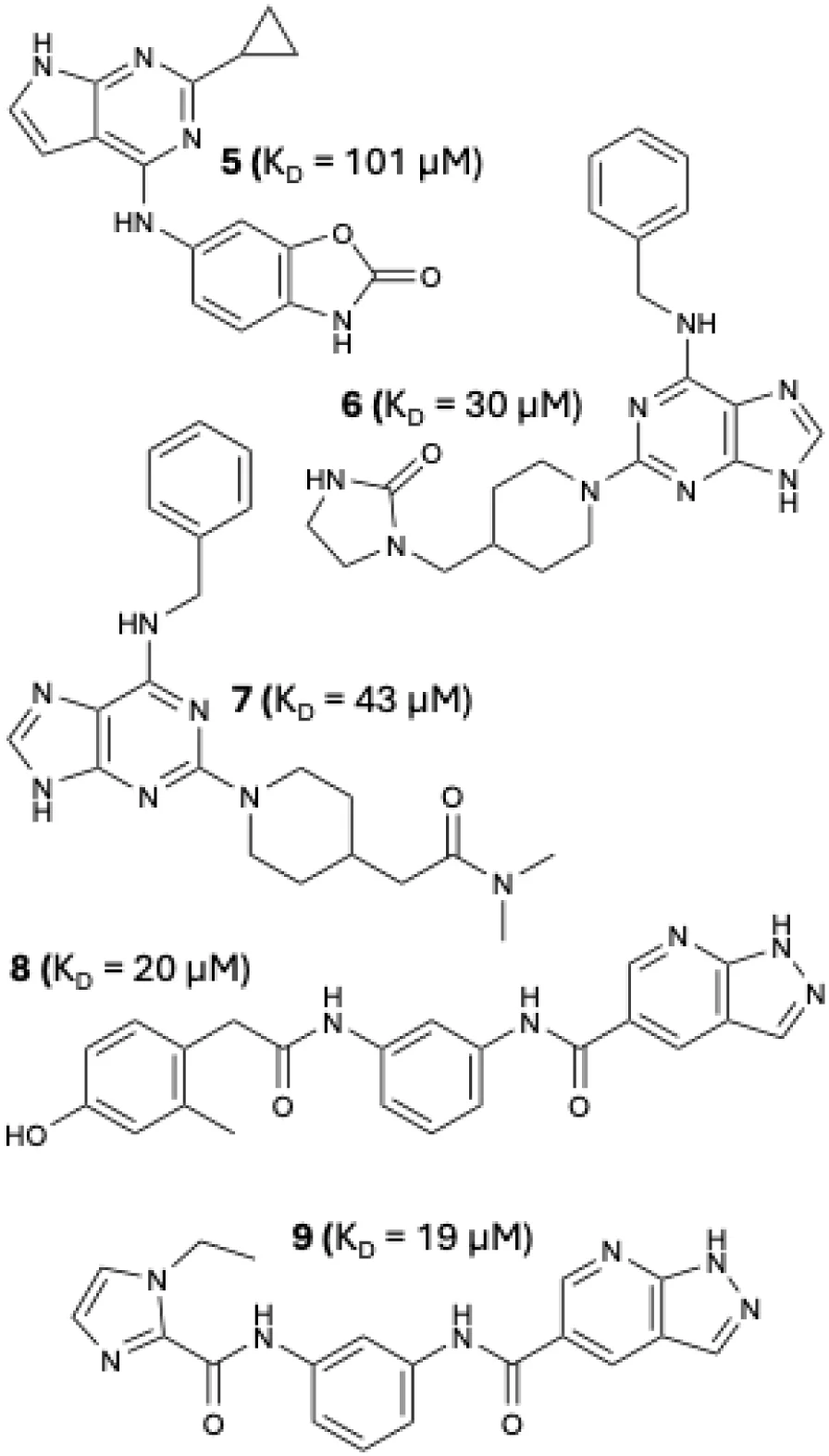
Experimentally confirmed CACHE #2 Round 2 hits. Chemical structures and *K_D_* values were determined by SPR.

## Discussion

Benchmarks like the CACHE Challenges^1,12,19^ provide a necessary reality check for *in silico* drug discovery. The logistical and financial demands of blindly validating submissions are justified by the high fidelity of the resulting data, which is free from the biases inherent in the retrospective studies. This rigorous framework advances the field by highlighting the most robust methodologies and facilitating access to the open-source tools. Furthermore, it directly contributes to the development of tangible chemical probes for complex biological systems such as the NSP13 helicase.

The SARS-CoV-2 NSP13 RNA-binding groove is the third challenging ligand-orphan target successfully addressed by FRASE-bot. Initially, the concept of fragments in structural environments was introduced to optimize selectivity profiles in a model system of three kinases (TYRO3, AXL, and MERTK).^10^ Subsequently, the method was applied to the Calcium and Integrin Binding protein 1 (CIB1)^9^ and the LRRK2 WDR domain.^11,12^ While CIB1 had known peptide binders that hinted at a binding site, both LRRK2 WDR and the current target, NSP13, represented genuinely orphan targets with no known small-molecule ligands. For NSP13, as with LRRK2 WDR, both the druggable pocket and the ligands needed to be identified simultaneously. The success of our integrated workflow on NSP13 suggests that these previous results were not a lucky strike but rather evidence of the method’s consistent ability to unlock difficult targets when combined with rigorous downstream filtering. While the CACHE Challenge format precludes a retrospective deconvolution of each computational step’s exact contribution, it is important to acknowledge that our protocol’s success relied on a synergistic combination of FRASE-based space delimitation, unsupervised clustering, and molecular docking. Nevertheless, because FRASE-bot has served as the common initial step across these multiple successful campaigns while utilizing varying subsequent filtering protocols, we posit that its ability to narrow the ultra-large chemical space to target-relevant compounds is a critical driver of the overall success rate.

In the CACHE Challenge #2,^19^ the performance of FRASE-bot was evaluated against 22 other computational workflows in a blind search for SARS-CoV-2 NSP13 ligands. The difficulty of the target was evidenced by the fact that only ten participants successfully identified active compounds. Across two rounds, our protocol yielded 4 experimentally confirmed binders from a total of 79 submitted candidates in Round 1, resulting in a 5% hit rate (as compared to the 2% average hit rate in CACHE #2). Surface Plasmon Resonance assays confirmed the affinities of these hits and their Round 2 analogs with *K*_*D*_ values ranging between 11 μM and 101 μM.^20^ These results underscore the efficacy of FRASE-based strategies for hit-finding against challenging, nonconventional targets. Fundamentally, this approach predicts ligand activity by analyzing the similarity of protein–ligand interaction patterns. While methods like SPLIF^21,22^ apply this concept to whole molecules, FRASE screening^9^ extends it to a fragment-wise analysis.

Furthermore, this study demonstrates that FRASE-bot provides a practical solution for navigating ultralarge chemical spaces. While libraries like Enamine REAL offer access to tens of billions of synthesizable compounds, they remain largely out of reach for standard virtual screening workflows, such as docking or 3D pharmacophore searches, due to the immense computational costs. FRASE screening overcomes this limitation by generating a set of privileged ligand fragments that define the necessary interaction features of the target. These fragments serve as queries for fast substructure searches, enabling the conversion of a generic, ultralarge library into a focused, target-specific chemical space. This process effectively bridges the gap between massive chemical availability and computational feasibility, allowing high-fidelity scoring methods to be applied exclusively to the most promising regions of the chemical universe.

Finally, the identification of validated binders targeting the RNA-binding groove of SARS-CoV-2 NSP13 holds significant promise for antiviral drug discovery. Unlike the rapidly mutating spike protein, the NSP13 helicase is highly conserved across the Coronaviridae family, making it an ideal target for pancoronavirus inhibitors that remain effective against emerging variants. The chemical matter identified in this study provides confirmed starting points for the development of potent chemical probes and potential therapeutics. These tools will be instrumental in dissecting the precise role of the helicase in the viral replication complex and paving the way for novel antiviral regimens that complement existing protease and polymerase inhibitors. We acknowledge that extended structural, biophysical, and biological characterization, such as high-resolution structural biology or orthogonal mechanistic assays, represents a critical next step for the further development of these hits. Although such comprehensive validation falls outside the scope of this initial hit-finding campaign, the primary objective of this work remains the rigorous benchmarking of computational hit finding against a highly challenging target. By providing experimentally validated starting points, this study demonstrates the robust potential of FRASE-bot and establishes a foundation for future collaborative optimization.

## Methods

### Computational Methods

*FRASE screening* was performed as described in ref 9 using NSP13 structure (PDB: 5RLH^5^) as the protein target. This specific structure was arbitrarily chosen as a representative model because the available CACHE structures for this target (5RLH, 5RLZ, 5RML, and 5RMM) are virtually identical in their overall conformations and binding site geometries. The workflow (including the postscreening cluster analysis by *k*-means method) was implemented in Pipeline Pilot 2021SP1 (available online^23^). Cartesian coordinates of the fragments’ geometric centers were used as input for *k*-means clustering (with the distance threshold of 2 Å for the cluster members).

*Substructure Search* for Round 1 was performed on the Enamine REAL database collection (downloaded in November 2022)^20^ using the RDKit function *rdkit.Chem.rdchem.HasSubstructMatch*

*Pharmacophore screening* was performed using the Phase module^16^ of the Schrödinger Suite Release 2021.^24^ Phase was applied in “full match” mode with a target number of 50 generated conformers per ligand and tolerance spheres of 1 Å. The Phase Screen Score was used for ranking the search hits with default rejection criteria.

*Ligand preparation.* The Enamine REAL 6.5 billion compound set was downloaded in November 2022 in SMILES format.^20^ All input files were standardized using a custom RDKit-based^22^ workflow. First of all, each structure underwent initial cleanup with the function *rdMolStandardize.Cleanup* which removes explicit hydrogens, disconnects metal atoms, normalizes functional groups, and corrects common valence states. If multiple fragments were present, then only one primary fragment was retained using *rdMolStandardize.FragmentParent*. The resulting molecules were then neutralized with RDKit’s built-in *rdMolStandardize.Uncharger* and converted to a canonical tautomer using *rdMolStandardize.TautomerEnumerator*.*Canonicalize.* Neutral sp^3^-hybridized amines were protonated and carboxylic acids were deprotonated. After these transformations, each molecule was sanitized by the *Chem.SanitizeMol.* The final standardized structures were exported as canonical isomeric SMILES. Duplicates were removed via the tool RDKitRemoveDuplicateMolecules.py from the MayaChemTools package.

For molecules with predefined stereocenters, the specified stereochemistry was preserved during the 2D-to-3D conversion. For molecules with undefined or racemic stereocenters, a single random enantiomer was generated by the 3D conversion function and retained, maintaining a single three-dimensional structure per compound without further stereochemical or conformational enumeration. This simplified strategy was adopted to control the computational burden of screening large chemical libraries. Enumerating all possible stereoisomers, tautomers, and ionization states per ligand would cause an exponential explosion in the compound volume, rendering the scale of screening campaigns computationally intractable. This simplified approach can result in false negatives if an active stereoisomer or alternative binding pose is missed, but we consider this to be an acceptable trade-off for keeping the workflow simple and minimizing the overall computational load.

*Ligand filters* included the following: (1) Modified Lipinski Rule of 5 (molecular weight between 300 and 600 Da, Log P between 1 and 5, number of (*N*) hydrogen bond donors ≤5, *N* hydrogen bond acceptors ≤12, and *N* rotatable bonds ≤12); (2) Structural filters, such as *N* phosphorus atoms = 0, *N* chiral centers <3, *N* rings >0, *N* aliphatic chains longer than 6 atoms = 0, *N* fused-ring systems with more than 4 rings = 0, *N* acyclic nitrogen–nitrogen bonds = 0, and *N* carboxyl groups + *N* amino groups ≤1, and (3) the removal of unwanted substructures. Unwanted substructures were defined as reactive, toxic, or historically assay-interfering functional groups, utilizing structural rules consistent with the established Rapid Elimination Of Swill (REOS) criteria.^15^

*Ligand-protein Docking* was performed using AutoDock Vina^17^ and Glide.^18^ The Vina program version 1.2.0 was used with the Vinardo scoring function and a search space of 40 × 40 × 40 Å centered on the geometric center of the 25 residues lining the RNA-binding groove of NSP13. These 25 residues were explicitly defined as those in close contact with the cocrystallized and FRASE-based ligand fragments selected as described in the Results section. The expanded volume of the box was explicitly chosen to ensure that all spatially distinct subpockets defined by the cocrystallized and FRASE-based fragments were fully encompassed within the box. All other parameters, including exhaustiveness, were kept at default values as we did not have any specific data suggesting a need to alter them, while the default parameter set had proven effective in our previous screening campaigns. The potential caveat of this choice is that it may lead to conformational under-sampling, potentially increasing the false-positive and false-negative rates. To address the false-positive risk, we relied on the downstream visual inspection step to strictly retain only those poses that corresponded to the specific pockets identified upstream in the workflow. Glide (Schrödinger Suite Release 2021 ^24^) was applied in Standard Precision mode with the default scaling factor and enabled sampling of ring conformations. The center of the protein grid was defined as the centroid of the Workspace ligand (ligand fragments in Round 1 or the docking pose of the confirmed hits in Round 2). The size of the grid was automatically determined to dock ligands similar in size to the Workspace ligands. The maximum number of ligand poses to output was set to one for both docking programs. In Round 2, the grid was defined based on the docking poses of experimentally confirmed Round 1 parent hits.

*Clustering and diversity filter.* The post-docking hit analysis was performed using the Butina clustering method to group similar molecules, implemented as the RDKit function *rdkit.ML.Cluster.Butina.ClusterData*. The fingerprints for all selected post-docking molecules were generated using *rdkit.Chem.rdFingerprintGenerator.GetRDKitFPGenerator.GetFingerprint*. The similarity of two fingerprints was calculated by *rdkit.DataStructs.TanimotoSimilarity*. The threshold for the clustering was set to 0.25.

*Similarity Search* was performed using the RDKit function *Chem.DataStructsTanimotoSimilarity*, where fp1 and fp2 represent Morgan fingerprints generated with a radius of 2 using *Chem.AllChem.GetMorganFingerprint*.

### Experimental Methods

#### Surface Plasmon Resonance (SPR)

The assay was conducted using a Biacore 8K (Cytiva) at 20 °C. Biotinylated Nsp13_SARS2, with approximately 4900–5100 response units (RU), was immobilized onto the flow cell two of a streptavidin-conjugated streptavidin chip following the manufacturer’s protocol. The flow cell one served as a reference for subtraction for each channel. Compounds were initially dissolved in 100% DMSO to create 10 mM stock solutions, which were subsequently serially diluted (factor: 0.5) to obtain six concentration points in 100% DMSO. For the SPR run, these serially titrated compound stocks were diluted at a ratio of 1:50 in HBS buffer, containing Mg^2+^ (10 mM HEPES pH 7.4, 150 mM NaCl, 5 mM MgCl2, 0.03% (v/v) Tween 20) to achieve a final DMSO concentration of 2%. Binding experiments used multicycle kinetics with a contact time of 60 s and a dissociation time of 180 s at a flow rate of 40 μL/min at 20 °C. The *K*_*D*_ values were determined using steady-state affinity 1:1 binding with the Biacore Insight Evaluation software (Cytiva).

#### Dynamic Light Scattering (DLS)

The solubility of compounds was estimated by DLS that directly measures compound aggregates and laser power in solution. Compounds were serially diluted directly from DMSO stocks, and then diluted 50× into filtered 10 mM Hepes pH 7.4, 150 mM NaCl, 5 mM MgCl_2_, and 0.03% Tween 20 (2% DMSO final). The resulting samples were then distributed into 384-well plates (black with a clear bottom, Corning 3540), with 20 μL in each well. The sample plate was centrifuged at 3500 rpm for 5 min before being loaded into the DynaPro DLS Plate Reader III (Wyatt Technology).

#### ATPase Activity

The level of ATP consumed by Nsp13 was quantified by measuring the amount of remaining ATP using a luciferase-based assay as previously described.^26^ The inhibitory effects of compounds were assessed in a 384-well format (14 μL final volume) using reactions composed of 50 mM HEPES, pH 7.5, 5% glycerol, 5 mM magnesium acetate, 5 mM DTT, 0.01% Triton X-100, 0.01% BSA, 0.1 nM Nsp13, 3.5 nM 30b PolyT ssDNA, 2.5 μM ATP, and 2% DMSO. Samples containing DMSO only (no compounds) were used as a control. Reactions were started by the addition of substrate and incubated for 60 min at room temperature. Then, 10 μL of the reactions were transferred into 384-well white plates containing 10 μL luciferase reagent (Cat# V6712; Promega, Madison, WI, USA) and incubated for another 20 min at room temperature. Compounds that were followed up for dose–response experiments were tested using the same luciferase reagent, and the data were analyzed using GraphPad Prism 9.

## Supplementary Material







## Data Availability

The Supplementary Data sets 1 and 2 can be found in the Supporting Information. The Pipeline Pilot implementation of FRASE-bot used in the CACHE #2 study can be found at the GitHub repository https://www.github.com/kireevlab/FRASE-bot-Pipeline-Pilot (this version is not supported anymore). The current FRASE-bot implementation, as a Python code making use of public libraries such as RDKit,^25^ is available at https://www.github.com/kireevlab/FRASE-bot-RDKit.

## Notes

Figure S1: 3D pharmacophore geometries superposed with the binding pocket (PDF)
Supplementary Data Set 1 containing data for compounds submitted to Round 1 of the challenge (XLSX)
Supplementary Data Set 2 containing data for compounds submitted to Round 2 of the challenge (XLSX)
