# Supplementary material for: Targeting the RNA-Binding Site of SARS-CoV‑2 NSP13 by FRASE-bot in CACHE Challenge #2
Source: J Chem Inf Model. 2026 Aug 6;66(16):10318–25. doi: 10.1021/acs.jcim.6c00560 (PMC13508771; doi:10.1021/acs.jcim.6c00560)
Supplement: Supplementary file 1 [file ci6c00560_si_001.pdf]

Targeting the RNA-binding Site of SARS-CoV-2 NSP13 by FRASE-bot in CACHE Challenge #2

Xiaowen Wang[1], Akhila Mettu[1], Oleksandra Herasymenko[2], Madhushika Silva[3], Scott Houliston[3], Irene Chau[2], Pegah Ghiabi[2], Elisa Gibson[3], Sumera Perveen[2], Almagul Seitova[3], Matthieu Schapira[2,3,4], Dmitri Kireev[1,5*]

[1]University of Missouri, Department of Chemistry, Columbia, MO, 65211, USA

[2]Structural Genomics Consortium, University of Toronto, Toronto, Ontario M5G 1L7, Canada

[3]Princess Margaret Cancer Centre, University Health Network, Toronto, Ontario M5G 2C4, Canada

[4]Department of Pharmacology & Toxicology, University of Toronto, Toronto, Ontario, M5S 1A8, Canada

[5]Lead contact

[*]Correspondence: dmitri.kireev@missouri.edu

Supplementary figures

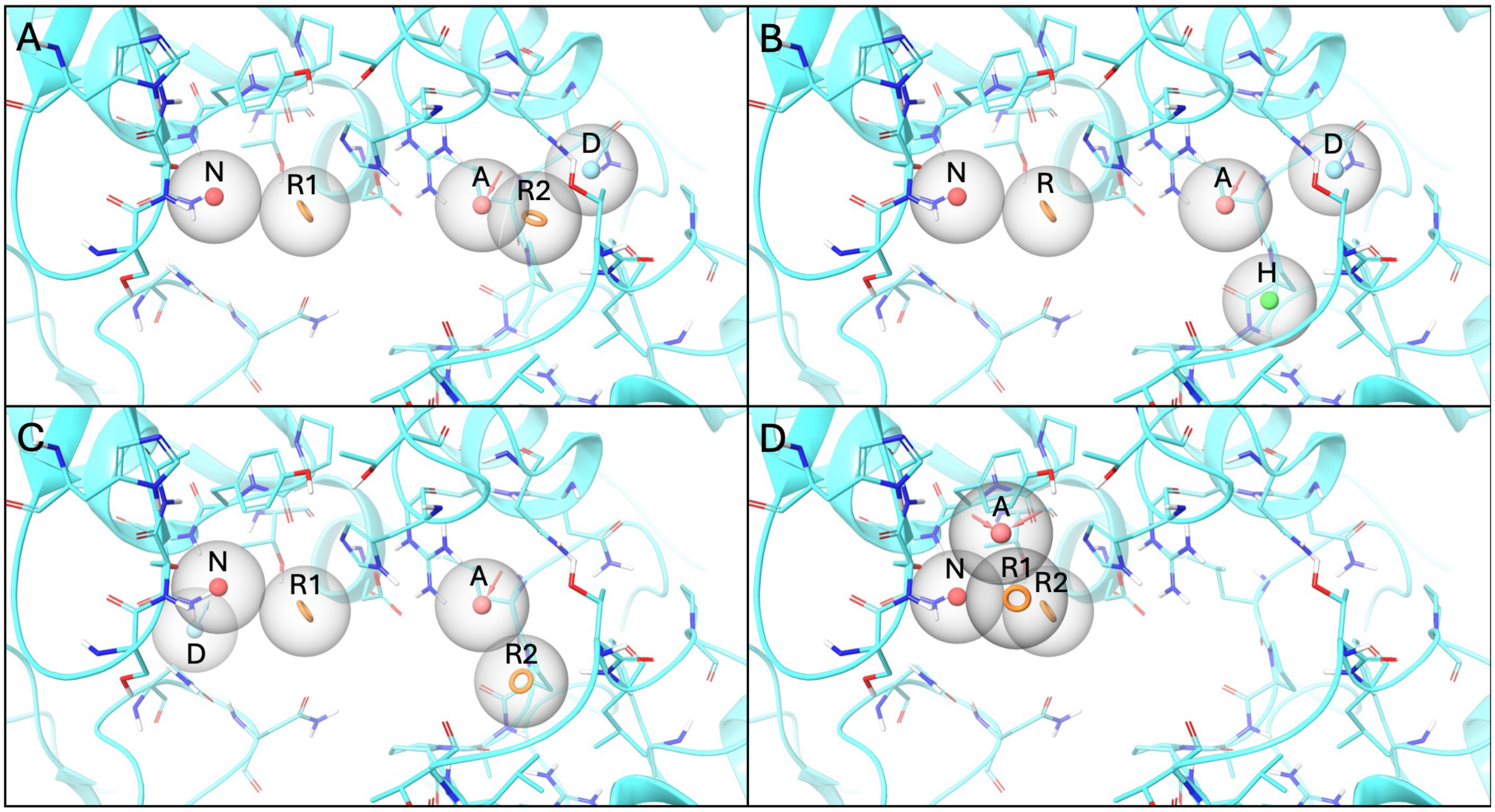

Supplementary Figure S1. 3D pharmacophore geometries superposed with the binding pocket. *(**A**) RRDNA(I), (**B**) RHDNA, (**C**) RRDNA(II), and (**D**) RRNA.*
